# Clinical and oncological outcomes of the low ligation of the inferior mesenteric artery with robotic surgery in patients with rectal cancer following neoadjuvant chemoradiotherapy

**DOI:** 10.3906/sag-2003-178

**Published:** 2021-02-26

**Authors:** İsmail GÖMCELİ, Orhan ARAS

**Affiliations:** 1 Department of Gastrointestinal Surgery, Antalya Training and Research Hospital, Health Sciences University, Antalya Turkey

**Keywords:** Rectal cancer, anastomosis stricture, inferior mesenteric artery, low ligation, robotic surgery, lymphadenectomy

## Abstract

**Background/aim:**

The aim of this study is to compare clinical and oncologic outcomes of the high and low ligation techniques of the inferior mesenteric artery (IMA) in rectal cancer patients treated with robotic surgery after neoadjuvant chemoradiotherapy (nCRT).

**Materials and methods:**

In this retrospective study, 77 patients with T3/T4-node negative rectal cancer with tumor penetration through the muscle wall (Stage 2) or node positive disease without distant metastases (Stage 3) who were treated electively with robotic surgical resection following nCRT at a single institution between January 2014 and January 2018 were analyzed. Patients were divided into 2 groups (38 patients were included in the low ligation group and 39 patients in the high ligation group).

**Results:**

There was no statistical difference between the high ligation group and low ligation group in univariate analysis for 2-year overall survival and disease-free survival (OR = 1.146; 95% CI = 0.274 to 4.797; P = 0.950, and OR = 1.141; 95% CI = 0.564 to 2.308; P = 0.713, respectively). There was no significant difference between the 2 groups in the mean number of harvested lymph nodes and mean number of metastatic lymph nodes (P = 0.980 and P = 0.124, respectively). Anastomosis stricture was observed significantly less frequently in the low ligation group versus the high ligation group (2.6% and 28.2%, respectively) (P = 0.002). Also, the difference for the median length of hospital stay for the high and low ligation groups was statistically significant in favor of the low ligation group (P = 0.011).

**Conclusion:**

In robotic rectal surgery, the low ligation technique of the IMA can reduce the rate of anastomosis stricture and provide similar oncological results as the high ligation technique.

## 1. Introduction

Colorectal canceris the 3rd most common cancer and the 4th most common cause of cancer-related deaths in the world [1]. Local recurrence rates for rectal cancer have even been reported as 20%–45% worldwide[2]. Several advances in surgical techniques such as total mesorectal excision (TME) reported by Heald and Ryall [3] and segmental resection of the tumor along with the en bloc associated lymphatic and vascular supply have remarkably improved clinical outcomes in patients with rectal cancer over the past decades [4]. The understanding of “en bloc lymph node resection to the origin level of the primary supply vessels” has been accepted as an important stage in this treatment [5]. More specifically, ligation of the root of the inferior mesenteric artery (IMA) proximal to the left colic artery (LCA) bifurcation (high ligation) is considered mandatory for wide lymph node dissection[6]. The rational for this is that it allows more adequate lymph node resection due to more extensive dissection around the root of the IMA. An extensive mobilization of the left colon may be more possible by the transection of the IMA at the root of abdominal aorta and can allow a tension-free and safer colorectal anastomosis. However, there is no consensus on the optimal level of ligation [4]because no difference in oncologic outcomes has been observed yet between high (proximal to the LCA bifurcation) and low (distal to the LCA bifurcation) ligation of the IMA[7,8].

However, some negative clinical results caused by high ligation are also mentioned in the literature. Hinoi et al. suggested that the high ligation of IMA in the laparoscopic abdominal resection for middle and low rectal cancer is associated with higher anastomotic leak rates [9]. Komen et al. also claimed, by showing higher blood flow rates in the low ligation group, that anastomosis would be better perfused in this group [10]. In addition, Sciuto et al. mentioned that reduced blood flow raises concern for colonic ischemia and an increased risk of anastomotic leak [11], but there are conflicting observations between studies. Some studies have mentioned early morbidities such as anastomosis leakage. However, long-term clinical results have not been mentioned, and patients could not be standardized for surgical procedures, tumor stages, tumor localizations, and neoadjuvant therapies.

Since the first robotic colon surgery was performed by Weber et al. [12], several reports have presented more beneficial clinical and oncological outcomes of robotic surgery for rectal cancers [13–16]. Thus, robotic systems have started to be used more widely, especially in minimally invasive colorectal surgery. Robotic surgery systems (da Vinci Surgical System, Intuitive Surgical Inc., Sunnyvale, California, CA, USA) have 3-dimensional (3D), enhanced high definition vision. In addition, the EndoWrist instruments (Intuitive Surgical, Inc.) of the system, the surgeon-controlled camera platform, and stable traction provided by the robotic arm provide surgeons with a range of motion far superior to that of the human hand and thus help surgeons to perform a more precise dissection in the pelvic cavity.

This study was designed to present the postoperative complications and 2-year survival rates of 77 consecutive patients with rectal cancer who underwent neoadjuvant chemoradiotherapy (nCRT) and robotic surgery with the high ligation and low ligation of the IMA.

## 2. Materials and methods

### 2.1. Patients selection and characteristics of the study

**Figure 1:**
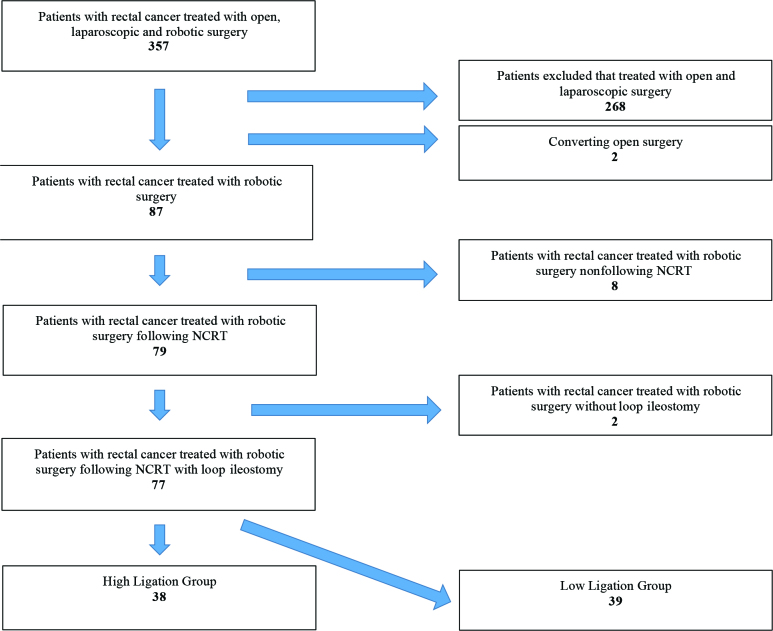
Election of the study groups.

In this retrospective study, we evaluated 357 consecutive patients with rectal cancer who underwent open, laparoscopic, and robotic surgery. We only included rectal cancer patients who underwent robotic TME resection with the da Vinci XI Surgical System following nCRT at a single institution between January 2014 and January 2018. Finally, we identified 77 patients treated electively with robotic surgical resection of rectal cancer following nCRT with primary anastomosis and loop ileostomy (Figure 1). 

All patients underwent a preoperative colonoscopic evaluation to determine the localization of the tumor and also abdominal and pelvic computed tomography (CT) or magnetic resonance imaging (MRI) for preoperative staging. None of the patients had preoperative whole gut cleaning. Patients received only 2 rectal enemas in the morning of the surgery.

Baseline covariates of the patients included age, sex, comorbidities, American Anesthesiology Association (ASA) classification, postoperative complications, previous abdominal surgical interventions, and length of hospital stay.

Patients with T3/T4-node negative rectal cancer with tumor penetration through the muscle wall or node positive disease without distant metastases had combined-modality therapy. nCRTwas performed as long course radiotherapy at 50.4Gy, concurrent with a fluoropyrimidine-based radiosensitizing chemotherapeutic agent (capacitabine or 5-FU). One or two cycles of induction chemotherapy after nCRT were performed in a period of 8 to 10 weeks until surgery. Induction chemotherapies were CapOx (capacitabineoxaliplatin) or FOLFOX (folinic acid-5-fluorouracil-oxaliplatin) and were followed by surgery.All patients received 6 cycles of adjuvant chemotherapy in the postoperative period.

Morbidities were defined as complications (e.g., anastomotic strictures, anastomotic leaks, surgical site infections, ileus, rectovaginal fistula, postoperative intraabdominal bleeding, uretheral injury, pulmonary embolism, and reoperation) that involved additional treatment or a prolonged hospital stay. Anastomotic stricture was defined as not allowing the passage of the colonoscope or allowing the passage but requiring a digital or balloon dilatation.

Tumour node metastasis (TNM) classification of the 8th edition of the American Joint Committee on Cancer (AJCC), tumor localization, number of harvested lymph nodes, number of metastatic lymph nodes, and the ratio of metastatic lymph node number to harvested lymph nodes were determined as tumor characteristics. 

Patients were treated by only 1 surgeon specialized in colorectal surgery. This surgeon was also trained and certified as an expert in the da Vinci Surgical System.

Patients were regularly followed up at an outpatient clinic. History taking and physical examination were conducted every 3 months for 1 year, then every 6 months for 2 years and, finally, on an annual basis. Control colonoscopy was performed in the 2nd month after adjuvant treatment was completed to close the loop ileostomy. Complications such as stricture, fistula, and dissociation in anastomosis were noted. If no problem was detected in the anastomosis, the loop ileostomy was closed. Abdominal and pelvic CT scans were performed annually for up to 3 years.The mean follow-up time for the high ligation group and the low ligation group was 33.03 ± 6.9 (range: 24–48) weeks and 33.76 ± 6.6 (range: 24–46) weeks, respectively.The study was approved by the local ethics committee (2020-2040).

### 2.2. Surgical procedure and ligation of the inferior mesenteric artery

The da Vinci Xi Surgical System was used for the surgical procedure and the 5-port technique was used for the docking method.Patients were grouped into 2 intervention groups: as the high ligation group if the IMA was divided proximal to the left colic artery bifurcation and in the low ligation group if the IMA was divided distal to the left colic artery bifurcation. The decision about the level of IMA ligation was made by the surgeon. The TME resection technique was performed in all patients. The robotic dissection for TME was as follows: a) the origin of the IMA from the abdominal aorta and the LCA bifurcation was explored. Lymphoadipose tissues around this area were skeletonized to perform complete D3 lymph node dissection, and the sympathetic nerve plexus surrounding the IMA was preserved; b) the LCA and superior rectal artery were identified and transected just distal to the bifurcation of the IMA and the LCA in the low ligation group; c) the IMA was transected at its origin from the aorta in the high ligation group; d) upward dissection of the mesentery along the ascending branch of the LCA was performed, and the inferior mesenteric vein (IMV) was clipped and transected in this area; e) medial to lateral dissection was performed, followed by colonic splenic flexure dissection to facilitate the complete mobilization of the descending colon; f) downward dissection of the presacral facia was performed until the cut reached the pelvic floor, with preservation of the paired hypogastric nerves and pelvic autonomic nerve plexus; g) the peritoneal reflection was incised laterally and followed anteriorly, and the rectum was separated circumferentially from the vagina/prostate up to the level of the levator muscles with the plane of Denonvilliers’ fascia; h) transection of the distal rectum was performed with a 60 mm endoscopic linear stapler.

The vascular supply of the proximal colon segment was macroscopically checked after the specimen was removed from the Pfannenstiel incision using an Alexis wound protector (Applied Medical Resources Corporation, California, CA, USA) to prevent wound infection in all patients. Transsection and anastomosis were performed after adequate blood supply was observed. Bowel continuity was reconstructed by end-to-end colorectal anastomosis with doublestapled technique by using a 31-mm EEA circular stapler (Medtronic Inc., Minneapolis, MN, USA). Anastomosis completeness was checked withthe air-water test and macroscopic examinations of donuts were made with a circular stapler in all patients. Diverting loop ileostomy was performed on all patients.

### 2.3. End points

The end points were made up of the postoperative anastomotic stricture rate, overall morbidity, postoperative morbidity, the length of hospital stay, the number of harvested lymph nodes, the number of metastatic lymph nodes, the rate of metastatic lymph nodes to harvested lymph nodes, the 2-year overall survival (OS) and disease-free survival (DFS) rates, and determination of factors affecting overall and disease-free survival.

### 2.4. Statistical analysis

Statistical analysis was made using IBM’s SPSS Statistics for Windows, Version 23.0 (IBM Corp., Armonk, Newyork, NY, USA). Data are expressed as n(%), mean±standard deviation (range) or median (range), as appropriate. Normality assumptions were controlled by the Shapiro–Wilk test. Categorical data were analyzed by the Pearson chi-square test or Fisher’s exact test. The differences between the 2 groups were evaluated with a Student’s t-test for normally distributed data or with the Mann–Whitney U test for nonnormally distributed data. OS and DFS were estimated using the Kaplan–Meier method. The log-rank test was used to compare survival differences. A univariate Cox proportional hazards regression model was used to identify prognostic factors. The variables which showed significant association with OS or DFS in the univariate analyses were further tested in the multivariate model. The hazard ratio, with corresponding 95% confidence intervals (95% CIs), was reported. A P-value of less than 0.05 was considered statistically significant. 

## 3. Results

### 3.1. Patients characteristics

Grouped as 38 patients in the low ligation group and 39 in the high ligation group, the clinical and pathological data of the 77 patients with rectal cancer who underwent nCRT followed by robotic surgery are summarized in Table 1. The median age of patients in the high ligation group and low ligation group was 61.6 years (range: 35–79 years) and 62.5 years (range: 43–82 years), respectively. In the high ligation group, 25 of the patients were male (64.1%) and 14 (35.9%) were female. In the low ligation group, 21 of the patients were male (55.3%) and 17 were female (44.7%). Six patients (15.4%) in the high ligation group and 7 patients (18.4%) in the low ligation group had previous abdominal surgery. Twenty-four patients (61.5%) in the high ligation group (17 hypertension with and/or 4 with diabetes mellitus) and 19 (50%) in the low ligation group (15 with hypertension and/or 11 with diabetes mellitus) had comorbidities. Preoperative nCRT was administered to all of the patients because all patients had stage 2 or 3 locally advanced rectum tumors during the preoperative period. There were no statistically significant differences between the high ligation group and the low ligation group regarding age, sex, previous abdominal surgery, nCRT, and comorbidities. However, diabetes mellitus was more common in the low ligation group (28.9% versus 10.3%), and the difference between the 2 groups was statistically significant in the subgroup analysis (P = 0.038).Mean time interval difference between nCRT completion and the robotic surgery in the high ligation group and the low ligation group was not significant (61 days and 60 days, respectively) (P= 0.495).

**Table 1:** Characteristics of all patients in the study.

	High ligation (n: 39)	Low ligation (n: 38)	P
Age (median) (range)	61.6 ± 10.8 (35–79)	62.5 ± 8.9 (43–82)	0.705
Sex Male/female	25 (64.1%)/14 (35.9%)	21 (55.3%)/17 (44.7%)	0.429
Previous abdominal surgery	6 (15.4%)	7 (18.4%)	0.722
Comorbidities	24 (61.5%)	19 (50%)	0.308
HT	17 (43.6%)	15 (39.5%)	0.714
DM	4 (10.3%)	11 (28.9%)	0.038
Neoadjuvant CRT	39(100%)	38(100%)	0.999
Time interval between NCRT completion androbotic surgery (day, mean) (range)	61(56-66)	60(57–63)	0.495
ASA			
I	14 (35.9%)	19 (50%)	0.211
II	25 (64.1%)	19 (50%)	0.136
Length of hospital stay (median)	6 (4–16)	5(4–17)	0.011
T Stage (yp)			
≤2	18 (46.2%)	20 (52.6%)	0.570
>2	21 (53.8%)	18 (47.4%)	0.295
N Stage (yp)			
0	27 (69.2%)	20 (52.6%)	0.324
1	7 (17.9%)	11 (28.9%)	0.528
2	5 (12.8%)	7 (18.4%)	0.356
AJCC Stage (yp)			
0	3 (7.7%)	1 (2.6%)	0.301
1	13 (33.3%)	13 (34.2%)	0.518
2	11 (28.2%)	6 (15.8%)	0.437
3	12 (30.8%)	18 (47.4%)	0.217
Tumor localization from anal verge (cm)	8 (5–11)	8 (4–11)	0.876
Number of harvested lymph node (mean) (range)	14.10 ±3.2 (8–22)	14.08 ± 4.9 (5–25)	0.980
Number of metastatic lymph node (mean) (range)	1.08 ± 2.2/0(0-8)	1.79 ± 3.1/0 (0–13)	0.124
Metastatic lymph node / harvested lymph node ratio	7%	10%	0.135

The ASA scores of all patients were I and II. The number of patients with ASA I in the high and low ligation groups was 14 (35.9%) and 19 (50%), respectively. The number of the patients with ASA II in the high ligation group and the low ligation group was 25 (64.1%) and 19(50%), respectively. The ASA scores of the high ligation group and low ligation group were similar (P = 0.211 for ASA I and P = 0.136 for ASA II). Median length of hospital stay was 6 (4–16) days for the high ligation group and 5 (4–17) days for the low ligation group. The difference was statistically significant in favor of the low ligation group (P = 0.011).The mean followup time for the high ligation group and the low ligation group was similar [33.03 ± 6.9 (range: 24–48) weeks and 33.76 ± 6.6 (range: 24–46) weeks, respectively; P = 0.854]. 

### 3.2. Association of level of ligation with postoperative complications

The postoperative complications in groups are summarized in Table 2. Postoperative complications were detected in 19 patients [12 (30.8%) in the high ligation group and 7 (18.4%) in the low ligation group]. The difference was also not statistically significant (P = 0.209). In the subgroup analysis, the differences were not significant for anastomosis leak, rectovaginal fistula, surgical site infection, postoperative bleeding, ureteral injury, postoperative ileus, and pulmonary embolism (P = 0.999, P = 0.240, P = 0.999, P = 0.999, P = 0.999, P = 0.999, and P = 0.494, respectively).

**Table 2:** Postoperative complications in the high ligation and the low ligationgroups.

	High ligation(n: 39)	Low ligation(n: 38)	P
Postoperative complications	12 (30.8%)	7 (18.4%)	0.209
Anastomosis stricture	11 (28.2%)	1 (2.6%)	0.002
Anastomosis leak	3 (7.7%)	2 (5.3%)	0.999
Rectovaginal fistula	3 (7.7%)	0 (0%)	0.240
Surgical site infection	3 (7.7%)	2 (5.3%)	0.999
Postoperative bleeding	1 (2.6%)	1 (2.6%)	0.999
Ureteral injury	1 (2.6%)	0 (0%)	0.999
Postoperative ileus	1 (2.6%)	0 (0%)	0.999
Pulmonary embolism	0 (0%)	1 (2.6%)	0.494

However, in the high ligation group, anastomosis stricture was observed in 11 (28.2%) patients in the case of colonoscopies performed before the closure of loop ileostomy, while anastomosis stricture was observed in 1 (2.6%) patient in the low ligation group. The difference was statistically significant (P = 0.002).

Six of the 11 anastomosis stricture patients in the high ligation group were treated with colonoscopic balloon dilatation, one patient was treated with digital dilatation, and 4 patients needed surgical intervention. Stapled anastomosis could be applied to 1 of the 4 patients who underwent surgery, while resection anastomosis could not be done to the other 3 due to the long segment of the stricture line so, in the end, a colostomy had to be performed. In the low ligation group, 1 patient with an anastomosis stricture wastreated with digital dilatation. Rectovaginal fistulas that developed in the high ligation group were observed on the 23rd, 35th, and 50th day following surgery. Anastomosis stricture was also observed in 2 of these patients. After approximately 6 months of follow-up, surgical treatment was needed due to lack of improvement, despite all nonsurgical treatments (such as discontinuation of oral intake and postponement of loop ileostomy closure).

In the high ligation group, 1 of the 3 patients with anastomosis leakage had undergone an end colostomy on the 7th postoperative day, and 1 of the patients was treated with primary suturing. In the other patient, anastomosis leakage was treated with the interruption of oral feeding without the need for surgical intervention. In the low ligation group, 1 of the 2 patients with anastomosis leakage had undergone an end colostomy on the 9th postoperative day, and the other one was treated with the interruption of oral feeding without the need for surgical intervention. Ureteral injury in the high ligation group was treated with the double-J ureteral stent placement.All other postoperative complications were treated with medical treatment without the need for surgical intervention. 

### 3.3. Association of level of ligation with tumor characteristics

Tumor characteristics of the groups were compared (Table 1). The distances of the tumors from the anal verge during preoperative colonoscopic assessment were similar in the 2 groups (8 cm in both groups).The mean number of harvested lymph nodes in the high ligation group was 14.10 ± 3.2 (range: 8–22) and 14.08±4.9 (range: 5–25) in the low ligation group. There was no significant difference between the 2 groups (P = 0.980). The difference for the mean number of metastatic lymph nodes was not statistically significant either in the high or the low ligation group (P = 0.124) (1.08 (range: 0–8) and 1.79 (range 0–13), respectively). The difference between the metastatic lymph node to harvested lymph node ratio between the 2 groups was not statistically significant (7% vs. 10%, P = 0.135). The tumor stages of the 2 groups did not differ statistically, and their T and N stages were equivalent, as well.

### 3.4. Survival benefit outcomes

OS was defined as the length of time from the operation to death, and DFS was defined as the time from operation to disease recurrence. 

**Figure 2:**
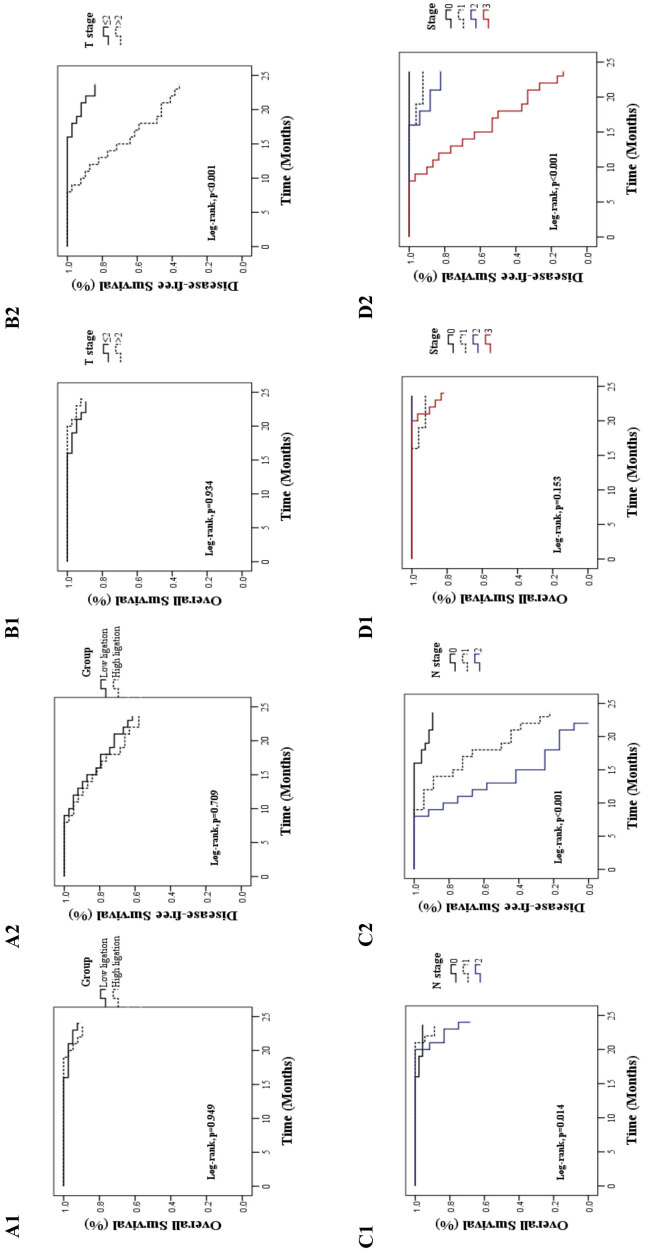
Kaplan–Meier estimates of overall survival and disease-free survival for the low and high ligation groups (A1, A2) for invasion status of tumor “T” (B1, B2), for lymph node status of tumor “N” (C1, C2), and for tumor stages (D1, D2).

Survival outcomes and univariate/multivariate analyses of factors affecting the survival outcomes were compared (Tables 3 and 4, Figure 2). No deaths occurred within 30 days in either group. It was determined that sex, previous abdominal surgery, ASA score, and length of hospital stay were not statistically significant in terms of 2-year OS and DFS. However, in the univariate analysis of the patients’age, it was observed that it did not affect disease-free survival, while it did affect overall survival (OR = 1.244; 95% CI = 1.099 to 1.41; P = 0.001). In the subgroup analysis of comorbidities, hypertension was found to be a determinant of overall survival (OR = 11.067; 95% CI = 1.361 to 90.02; P = 0.025). Similarly, only postoperative bleeding was found to be a determinant of disease-free survival in the univariate subgroup analysis of postoperative complications (OR = 9.779; 95% CI = 2.173 to 44.011; P = 0.003).After analyzing pathological data from all rectal cancers, our results showed that all T3, T4, N1, N2, and stage 3 and above tumors significantly determined DFS. However, only N2 tumors were found to affect OS (Table 3).

**Table 3:** Univariate analysis for OS and DFS (OS: overall survival; DFS: disease-free survival).

	OS		DFS	
	HR (95%CI)	P	HR (95%CI)	P
Age	1.244 (1.099–1.41)	0.001	1.016 (0.977–1.056)	0.424
Sex				
Female	Reference	-	Reference	-
Male	1.146 (0.274–4.797)	0.852	1.571 (0.74–3.337)	0.240
Study groups				
Low ligation	Reference	-	Reference	-
High ligation	1.146 (0.274–4.797)	0.950	1.141 (0.564–2.308)	0.713
Previous abd. surgery	1.78 (0.359–8.825)	0.480	1.256 (0.515–3.063)	0.616
Comorbidities	6.024 (0.741–48.981)	0.093	1.12 (0.549–2.286)	0.756
HT	11.067 (1.361–90.02)	0.025	1.707(0.844–3.455)	0.137
DM	2.71 (0.647–11.348)	0.172	1.425 (0.637–3.188)	0.388
Postop. Ccomplications	1.805 (0.431–7.555)	0.419	2.073 (0.993–4.329)	0.052
Anastomosis stricture	0.733 (0.09–5.957)	0.771	0.492 (0.15–1.619)	0.243
Anastomosis leak	0.045 (0–8632.021)	0.617	0.466 (0.064–3.422)	0.453
Rectovaginal fistula	3.891 (0.477–31.725)	0.204	1.45 (0.346–6.085)	0.611
Surgical Ssite infection	0.045 (0–86.021)	0.617	2.523 (0.765–8.323)	0.129
Postop. bleeding	5.111 (0.629–41.55)	0.127	9.779 (2.173–44.011)	0.003
ASA				
I	Reference	-	Reference	-
II	5.702 (0.701–46.363)	0.103	1.227 (0.595–2.527)	0.580
Length of hospital stay	0.847 (0.612-1.172)	0.316	1.038 (0.941–1.146)	0.455
T Stage				
≤2	Reference	-	Reference	-
>2	0.944 (0.236–3.773)	0.935	6.255(2.555–15.311)	<0.001
N Stage				
0	Reference	-	Reference	-
1	2.618 (0.369–18.588)	0.336	11.444 (4.087–32.045)	<0.001
2	8.392 (1.535–45.871)	0.014	32.31 (10.93–95.51)	<0.001
AJCC Stage				
≤2	Reference	-	Reference	-
>2	4.833 (0.975–23.96)	0.054	15.714 (5.96–41.436)	<0.001
Tumor localization from anal verge	0.776 (0.522–1.154)	0.210	1.061 (0.871–1.291)	0.557
Number of harvested lymph nodes	1.153 (0.985-1.351)	0.076	1.211 (1.11–1.321)	<0.001
Number of metastatic lymph nodes	1.213 (1.036–1.42)	0.017	1.255 (1.165–1.351)	<0.001
Metastatic lymph node/harvested lymph node ratio	1.036 (1.003–1.069)	0.030	1.051 (1.035–1.067)	<0.001

**Table 4:** Multivariate analysis for OS and DFS (OS: overall survival; DFS: disease-free survival).

	OS		DFS	
	HR (95%CI)	P	HR (95%CI)	P
Age	1.46 (1.127–1.89)	0.004	-	-
HT	7.06 (0.524–95.177)	0.141	2.18(0.914-5.201)	0.079
ASA				
I	Reference	-	-	-
II	0.034 (0.001–1.813)	0.096	-	-
Postop. Complications	-	-	2.330 (1.019–5.327)	0.045
T Stage				
≤2	-	-	Reference	-
>2	-	-	4.644 (1.669–12.918)	0.003
N Stage				
0	Reference	-	Reference	-
1	1.375 (0.156–12.137)	0.774	8.026 (2.663–24.185)	<0.001
2	0.76 (0.014–41.462)	0.893	9.393 (1.961–45)	0.005
Number of harvested lymph nodes	1.203 (0.962–1.505)	0.105	1.059 (0.93–1.207)	0.386
Number of metastatic lymph nodes	1.24 (0.707–2.174)	0.453	1.05 (0.892–1.234)	0.558

When the dissected lymph nodes were evaluated in the univariate analysis, the number of metastatic lymph nodes and themetastatic lymph node/harvested lymph node ratios were found to be significant for both OS and DFS. However, the number of harvested lymph nodes was significant only for DFS (Table 3).

There was no statistical difference between the high ligation group and the low ligation group in the univariate analysis for 2-year OS and DFS (OR = 1.146; 95% CI = 0.274 to 4.797; P = 0.950 and OR = 1.141; 95% CI = 0.564 to 2.308; P = 0.713, respectively). In the multivariate analysis of all factors that are significant in the univariate analysis, only patient age was found to be significant for OS. Postoperative complications, T3, T4, N1, and N2 tumors were found to be significant for DFS (Table 4, Figure 2).

## 4. Discussion

In rectal cancer surgery, studies comparing the clinical and oncological outcomes of open surgery, laparoscopic surgery, and robotic surgery are currently being carried out, and recent studies have reported the potentially significant benefits of robotic surgery for rectal cancer [17–24]. The current robotic operative system has the advantages of offering stable vision, 3-dimensional view, and superior dexterity and precision of the movements of the robotic arms. Hence, robotic surgery allows more precise dissection in the pelvic cavity, thus increasing the sphincter preservation rate, decreasing circumferential resection margin (CRM) positivity, and reducing the conversion rates in patients with low rectal cancer [25]. Similar advantages can even be gained in patients with rectum cancer receiving nCRT before surgery [26].

The main issue under discussion is the adequacy of oncological outcomes to be obtained as a result of minimally invasive surgery. A randomized trial, COREAN, which compared open versus laparoscopic surgery for mid to low rectal cancer after neoadjuvant chemoradiotherapy, revealed no difference in the rate of CRM positivity rates of completeness of mesorectal resection, and no difference in 3-year disease-free survival [27,28]. A randomized, international, multicenter study comparing the outcomes of laparoscopic and conventional resection of rectal carcinoma, The European COLOR II trial, resulted in similar safety, resection margins, completeness of resection, and 3-year locoregional recurrence and survival rates [29,30].

In some studies about robotic and laparoscopic surgery applications for rectal cancer, there was no difference in operation time, complication, and leak rates. The quality of the TME specimen was acceptable in both groups, and there were more complete specimens in the robotic group [31]. Moreover, in some studies, the conversion rate was significantly lower for the robotic group with a better DFS compared with the laparoscopic group [32].

Another issue is in regard to the completeness of TME, which was studied in a metaanalysis by Milone et al. in 2019. It was concluded in the analysis of 1520 procedures that completed TME showed a statistically significant difference in favor of robotic surgery [33].

Likewise, the number of harvested lymph node metastasis is a crucial factor in predicting the prognoses of colorectal cancer patients. At least 12 lymph nodes should be examined for each surgical specimen, as recommended in the AJCC/UICC guidelines. However, it is very difficult to reach this recommended number of lymph nodes after nCRT, and the number of harvested lymph nodes significantly decrease after preoperative nCRT with the median number of lymph nodes at 4 to 14 [34,35]. In this context, which level of IMA dissection must be performed in order to reach the recommended number of lymph nodes in colorectal cancer surgery has become a current issue. Consequently, the high or low ligation level of IMA remains controversial today. In many studies, it has been reported that high ligation of IMA will result in more satisfactory survival and adequate staging [36–38]. However, low ligation of the IMA with the preservation of the LCA has recently been suggested by some surgeons [39–41]. It has also been claimed that the high ligation technique can reduce blood flow in the colon and then cause intestinal ischemia, and that this eventually may lead to anastomosis leakage. Moreover, 5 retrospective cohort studies and 2 randomized clinical trials showed that the level of ligation had no impact on oncologic outcomes [8,42,43].

All factors such as open or minimally invasive surgery, the number of harvested lymph nodes, the number of metastatic lymph nodes, the level of IMA ligating, tumor stage, and nCRT appear to affect oncological and clinical outcomes. However, studies need to be standardized in order to evaluate these factors. However, laparoscopic and robotic surgery, patients with or without nCRT, and sigmoid or rectum cancers are evaluated together in most studies. In particular, performing a standard surgery is mandatory in terms of standard study results.

In this study, all parameters that may affect the study results tried to be standardized while evaluating the clinical and oncological results of IMA high or low ligation. For example, age, sex, previous abdominal surgery, comorbidities, diverting loop ileostomy application, nCRT status, time from nCRT to surgery, and ASA scores were all standardized. Additionally, a single surgeon experienced in robotic colorectal surgery performed all of the operations. Furthermore, in this study, there was no difference in the high ligation versus the low ligation group for the TNM stages, the number of harvested lymph nodes, the number of metastatic lymph nodes, and the ratio of metastatic lymph nodes to harvested lymph nodes.

When the clinical results of high and low IMA ligation were evaluated after the standardization of all of these clinical parameters, a significantly increased anastomosis stricture was observed in the high ligation group.

Although some studies suggest that the more extensive resection of mesenteric lymphatic drainage that is associated with high ligation increases the survival rate and reduces the recurrence rate [44–46], our study showed no significant differences with regard to 2-year OS and DFS between high or low ligation of the IMA, as has been shown in several other studies [47,48].

Some authors have suggested that the status of the lymph nodes around the IMA root is the most important determinant of DFS [49],but Adachi et al. analyzed lymph node metastasis distribution along the IMA and indicated that only 0.7% of the patients had a positive lymph node at the root of the IMA[50].Therefore, as in our study and in some other studies [51,52], adequate oncologic outcomes can be achieved by low ligating IMA with robotic rectal cancer surgery.

Studies comparing the high or low binding of IMA have generally evaluated anastomosis leakage as a study end point [9,40,53]. However, evaluation of stricture development in anastomosis may be an important endpoint, because the stricture, as in our study, may be due to the lack of blood flow that develops after the high binding of IMA. This has been shown with laser Doppler flowmetry inthe study of Komen et al. [54]. This lack of blood flow may also cause anastomosis leaks. In addition, it is also very difficult to manage these anastomosis strictures. Although some authors suggest the resection of this area with a circular stapler for the treatment of anastomosis strictures [55], this may not always be possible since stricture often includes a long colon segment, as seen in our 3 patients in the high ligation group; in addition, sometimes surgery could have to be terminated with an end colostomy, especially in anastomoses located below. Also, it should be observed that diversion colitis might contribute to the high anastomosis stricture rate (28.2%) in the high ligation group in our study. However, in order to standardize the cases, patients without diversion ileostomy were excluded from this study. Therefore, the effect of diversion colitis is valid for both groups. Moreover, the similar anastomosis leak rate between the 2 groups reveals more clearly the effect of anastomosis blood flow on the development of stricture because, even if anastomosis leakage does not develop, low blood flow in the high ligation group may be the cause of stricture in long-term follow-up.

It may be claimed that the high binding of IMA can ensure a tension-free colorectal anastomosis. However, such a problem can be resolved if the IMV is ligated at the level of the lower border of the pancreas, and if the lateral attachments of the descending colon are mobilized to the level of the splenic flexure, as Liang et al. have described [51].

Another approach for anastomosis safety is intraoperative assessment of perfusion at the site of anastomosis with indocyanine green (ICG). In some studies, more anastomosis leakage was observed in the group that was detected to have poor perfusion onfluorescence angiography via ICG [56–58]. However, Boni et al. compared patients undergoing low anterior resection with or without ICG angiography, and they observed no significant differences between the 2 groups in terms of the anastomosis leak.

In conclusion, in robotic low anterior resection,performed by experienced surgeons, the low ligation technique of the IMA can reduce the rate of anastomosis stricture and provide similar oncological results as the high ligation technique. 
